# The fragrance of sadness: Resisting the medicalization of human emotion in palliative care

**DOI:** 10.1017/S1478951526102016

**Published:** 2026-03-02

**Authors:** Raissa Milan-Youssef, João Carlos Geber-Júnior

**Affiliations:** 1 Faculty of Health Sciences, Sírio-Libanês Hospital, São Paulo, Brazil; 2 Center for Oncologic Intercurrence Care (CAIO), Cancer Institute of the State of São Paulo (ICESP), São Paulo, Brazil; 3 Hospital das Clínicas, Faculty of Medicine, University of São Paulo (FMUSP), São Paulo, Brazil

We choose to write about sadness because it is both ubiquitous in serious illness and persistently misunderstood in clinical settings. In contemporary healthcare, a lowering of mood is frequently interpreted as a sign of depressive disorder, and the implied appropriate response is often pharmacological. From a psycho-oncology perspective, however, sadness is commonly a proportionate emotional response to threatened meaning, anticipated loss, and profound uncertainty. Treating it primarily as pathology risks obscuring its human and relational significance.

This tension is particularly evident in palliative care, where diagnostic categories are never morally neutral. How clinicians interpret sadness shapes what they attend to, what they avoid, and which forms of care become conceivable. Qualitative work published in this journal has demonstrated that even among psychiatrists working in palliative care, “depression” is a heterogeneous and contested concept, encompassing diverse experiences that do not always map neatly onto psychiatric nosology (Ng et al. 2015). This finding invites caution: when sadness is rapidly translated into diagnosis, something essential may be lost.

The ethical stakes of this distinction were articulated decades ago by Eric Cassell, who argued that suffering arises not from disease alone, but from threats to the integrity of the person (Cassell 1982). Sadness, in serious illness, often signals precisely such a threat. It reflects disruptions in biography, identity, and imagined futures – dimensions that cannot be resolved through symptom control alone.

From the psychologist’s clinical experience in psycho-oncology, sadness is nearly universal. Over years of oncology practice, referrals for psychiatric assessment with the explicit aim of confirming depressive disorder and initiating medication were relatively infrequent. Sadness, however, was present in almost every encounter. The most consequential variable was not its intensity, but whether it could be shared.

One patient once explained that she attended therapy simply “to cry,” because it was the only place where she felt permitted to express her feelings without restraint. That sentence revealed a critical truth: suffering becomes most unbearable when sadness must be carried alone. This observation resonates with conceptualizations of loneliness as a subjective and painful emotional experience, independent of physical isolation. In palliative care contexts, loneliness has been described as affecting both patients and those who care for them, often persisting even in the presence of others (Rokach et al. 2007). More recent work has reinforced loneliness as a significant and under-addressed dimension of psychosocial suffering in cancer care (Gray et al. 2020).

From the physician’s palliative care perspective, this matters because modern clinical culture privileges action over accompaniment. When sadness resists resolution, it may provoke discomfort, leading clinicians to avoid it, normalize it prematurely, or medicalize it reflexively. Psychiatric literature has warned that diagnostic frameworks can blur the boundary between depressive disorder and proportionate sadness, contributing to the medicalization of ordinary human responses to adversity (Durà-Vilà and Hodes 2013). In palliative care, such blurring carries ethical consequences: it may inadvertently intensify isolation by signaling which emotions are acceptable within medical spaces.

Motivated by these concerns, we created a support group in the clinical setting where we work. The group was not conceived as a formal psychotherapeutic intervention, but as a relational space in which sadness could be expressed, witnessed, and shared – without urgency to categorize or suppress it. The first meeting included a symbolic dynamic that illustrates how we understand sadness and how we attempt to care for it.

Before participants entered the room, we placed a vase on the table filled with dry branches and leaves, without water. Each participant received a Gerbera flower as a gesture of welcome. We invited them to observe the vase and describe what it represented. Words such as “death,” “sadness,” and “ugliness” emerged frequently. After listening to all contributions, we added water to the vase and invited participants to introduce themselves – not through diagnosis but through story: where they lived, what they enjoyed, who they were beyond illness. At the end of each introduction, each person placed their flower into the vase.

The transformation was immediate.

We then discussed the dry branches as representations of difficult moments in life – experiences already woven into each participant’s biography. Importantly, the branches were not removed. We wanted to make visible a central insight of palliative care: hardship and beauty are not mutually exclusive. Diagnosis and the sadness it brings do not erase the value of a life trajectory. On the contrary, such moments often contribute to the development of emotional resources and maturity.

The water symbolized care. We emphasized that the purpose of the group was not to eliminate sadness but to tend to emotional life: to create a hospitable environment in which vitality and meaning – the flowers – could emerge and endure alongside the branches. In this sense, the group functioned as a relational intervention, aligned with a tradition in palliative care that recognizes the legitimacy of support groups as spaces for shared meaning and emotional containment (Henriksson et al. 2011).

From a broader palliative care perspective, this experience reinforces an understanding of care not as constant intervention, but as discernment. Not all suffering requires correction. Much suffering, however, requires presence. In prior work, we have argued that palliative care may be understood less as a referral triggered by prognosis and more as a clinical stance grounded in time, presence, and an honest acknowledgment of medicine’s limits (Geber-Junior and Forte 2025a). We have also explored how communication fails when it becomes a script rather than a relational act capable of holding uncertainty and personhood (Geber-Junior and Forte 2025b). The present reflection extends those arguments into the emotional domain: sadness is one of the most common expressions of threatened meaning in serious illness.

The most striking outcome of the group emerged weeks later, when participants organized a spontaneous meeting outside the scheduled sessions. This unplanned continuation suggested that something essential had occurred: sadness had become relational. Connection emerged not because sadness was removed, but because it was allowed to exist within a shared space. New buds appeared among the dry branches. If one could name this species of flower, perhaps it would be hope.

This perspective does not argue against psychiatric care or pharmacological treatment when clinically indicated. Rather, it calls for ethical and clinical discernment. Medical authority carries responsibility: to distinguish pathology from humanity, and intervention from accompaniment. In honoring sadness as a legitimate human response, clinicians affirm the dignity of patients as persons – precisely when illness threatens to reduce them to diagnoses alone.

Allowing sadness to be expressed, witnessed, and shared is not abandonment. It is care.
